# Oxidative Stress and Mitochondrial Abnormalities Contribute to Decreased Endothelial Nitric Oxide Synthase Expression and Renal Disease Progression in Early Experimental Polycystic Kidney Disease

**DOI:** 10.3390/ijms21061994

**Published:** 2020-03-14

**Authors:** Alp S. Kahveci, Tania T. Barnatan, Ali Kahveci, Alexis E. Adrian, Jennifer Arroyo, Alfonso Eirin, Peter C. Harris, Amir Lerman, Lilach O. Lerman, Vicente E. Torres, Maria V. Irazabal

**Affiliations:** 1 Department of Internal Medicine, Division of Nephrology and Hypertension, Mayo Clinic, 200 First Street SW, Rochester, MN 55905, USA; askahveci@gmail.com (A.S.K.); tania.barnatan@gmail.com (T.T.B.); kahveciali95@gmail.com (A.K.); aadrian@cord.edu (A.E.A.); arroyo.jenifer@mayo.edu (J.A.); eirinmassat.alfonso@mayo.edu (A.E.); harris.peter@mayo.edu (P.C.H.); lerman.lilach@mayo.edu (L.O.L.); torres.vicente@mayo.edu (V.E.T.); 2 Mayo Translational PKD Center, Mayo Clinic, Rochester, MN 55905, USA; 3 Department of Cardiovascular Diseases, Mayo Clinic, Rochester, MN 55905, USA; lerman.amir@mayo.edu

**Keywords:** polycystic kidney disease, oxidative stress, NOX4, mitochondria, endothelial dysfunction

## Abstract

Vascular abnormalities are the most important non-cystic complications in Polycystic Kidney Disease (PKD) and contribute to renal disease progression. Endothelial dysfunction and oxidative stress are evident in patients with ADPKD, preserved renal function, and controlled hypertension. The underlying biological mechanisms remain unknown. We hypothesized that in early ADPKD, the reactive oxygen species (ROS)-producing nicotinamide adenine dinucleotide phosphate hydrogen (NAD(P)H)-oxidase complex-4 (NOX4), a major source of ROS in renal tubular epithelial cells (TECs) and endothelial cells (ECs), induces EC mitochondrial abnormalities, contributing to endothelial dysfunction, vascular abnormalities, and renal disease progression. Renal oxidative stress, mitochondrial morphology (electron microscopy), and NOX4 expression were assessed in 4- and 12-week-old PCK and Sprague-Dawley (wild-type, WT) control rats (n = 8 males and 8 females each). Endothelial function was assessed by renal expression of endothelial nitric oxide synthase (eNOS). Peritubular capillaries were counted in hematoxylin–eosin (H&E)-stained slides and correlated with the cystic index. The enlarged cystic kidneys of PCK rats exhibited significant accumulation of 8-hydroxyguanosine (8-OHdG) as early as 4 weeks of age, which became more pronounced at 12 weeks. Mitochondria of TECs lining cysts and ECs exhibited loss of cristae but remained preserved in non-cystic TECs. Renal expression of NOX4 was upregulated in TECs and ECs of PCK rats at 4 weeks of age and further increased at 12 weeks. Contrarily, eNOS immunoreactivity was lower in PCK vs. WT rats at 4 weeks and further decreased at 12 weeks. The peritubular capillary index was lower in PCK vs. WT rats at 12 weeks and correlated inversely with the cystic index. Early PKD is associated with NOX4-induced oxidative stress and mitochondrial abnormalities predominantly in ECs and TECs lining cysts. Endothelial dysfunction precedes capillary loss, and the latter correlates with worsening of renal disease. These observations position NOX4 and EC mitochondria as potential therapeutic targets in PKD.

## 1. Introduction

Autosomal dominant polycystic kidney disease (ADPKD) is a systemic disorder where progressive development and enlargement of bilateral renal cysts lead to end-stage renal disease (ESRD). In addition, ADPKD is associated with multiple extrarenal manifestations involving the heart and vasculature, with cardiovascular complications being an important cause of morbidity and the main cause of mortality [1,2,3,4,5,6].

The endothelium is a key player in cardiovascular disease, and endothelial dysfunction, characterized by an imbalance between vasodilating, in particular nitric oxide (NO), and vasoconstricting substances acting on the endothelium, is one of the first events in cardiovascular disease [7,8]. Previous studies have shown that systemic markers of endothelial dysfunction and oxidative stress are evident in patients with ADPKD, preserved kidney function, and controlled hypertension (HTN) [9]. The primary biological mechanisms underlying oxidative stress and endothelial dysfunction in ADPKD remain to be fully elucidated.

The nicotinamide adenine dinucleotide phosphate hydrogen (NAD(P)H)-oxidase complex-4 (NOX4) and the mitochondrial respiratory enzymes are the major sources of endogenous reactive oxygen species (ROS) in both renal tubular epithelial cells (TECs) and endothelial cells (ECs) [10,11]. Importantly, there is an interplay between these two ROS sources that can lead to feed-forward mechanisms where the activation of one source of ROS can lead to the activation of another one (ROS-induced ROS production) [12]. Elevated NOX4 expression has been shown to increase mitochondrial ROS (mtROS) production, whereas its downregulation restores mitochondrial bioenergetics and reduces mtROS [13]. Increased mtROS can not only further instigate mtDNA damage, oxidation of mitochondrial proteins, and mitochondrial dysfunction, but also affect intracellular signaling pathways such as endothelial NO synthase (eNOS) uncoupling [14], promoting endothelial dysfunction [15].

Several recent studies in murine models of PKD have reported mitochondrial morphological and functional abnormalities in TECs lining cysts [16,17,18]. Yet, whether mitochondrial abnormalities are evident in non-cystic TECs and other parenchymal cells, such as ECs, remains unknown. Furthermore, whether NOX4 expression is altered in ADPKD has not been reported. Therefore, the objective of this study was to determine the main sources of ROS in early PKD and whether NOX4-induced oxidative stress results in EC mitochondrial abnormalities and contributes to endothelial dysfunction, vascular abnormalities, and renal disease progression.

## 2. Results

### 2.1. Animal Clinical and Laboratory Parameters

Table 1 shows the main animal characteristics of 4- and 12-week-old PCK and Sprague-Dawley (wild-type, WT) control rats (n = 16, 8 males and 8 females). Body weight was similar at 4 and 12 weeks in PCK and WT rats, but PCK animals had significantly enlarged kidneys already at 4 weeks, becoming more pronounced at 12 weeks. The cystic index, assessed from histological images, progressively increased from 4 to 12 weeks and extended from predominantly medullary cysts at 4 weeks to a more uniform distribution by 12 weeks (Supplementary Figure S1). Fibrotic index, on the other hand, was not different between PCK and WT rats until 12 weeks (Table 1). There was no difference in urine output at 4 or 12 weeks. Blood urea nitrogen (BUN) was similar at 4 weeks in PCK and WT rats but was higher in PCK rats at 12 weeks compared to WT animals. Contrarily, serum creatinine was not different at either 4 or 12 weeks. Blood pressure was similar between groups at 4 weeks. By 12 weeks, blood pressure levels became slightly, but not significantly, elevated in PCK compared to WT rats.

### 2.2. Renal Oxidative Stress Worsens with Disease Progression in PKD

**Figure 1:**
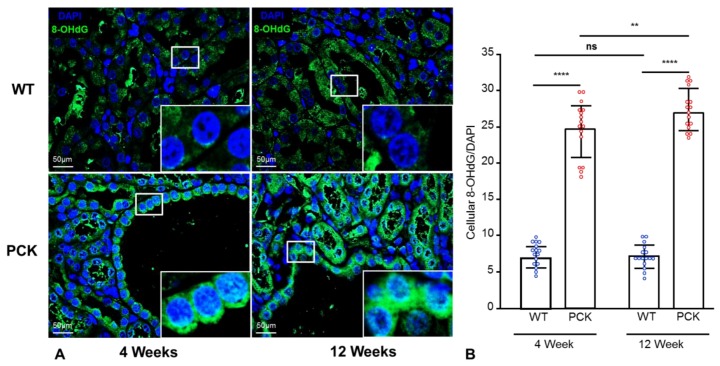
Renal oxidative stress worsens PKD progression. Representative immunofluorescence (IF) staining for 8-hydroxyguanosine (8-OHdG, green) in renal tissue sections of WT and PCK rats (**A**) and its quantification (**B**), showing increased 8-OHdG immunoreactivity in PCK vs. WT rats at 4 weeks, which further increased at 12 weeks; 8-OHdG was quantified as % stained area and adjusted to DAPI-stained % area. ** *p* < 0.01, **** *p* < 0.001. (n = 16/group).

To assess renal oxidative stress at early stages in PKD, we stained renal sections with 8-hydroxyguanosine (8-OHdG), an oxidized DNA damage byproduct and a surrogate marker of oxidative stress. Renal tubular cells from PCK animals exhibited a significant cytoplasmic and nuclear accumulation of 8-OHdG as early as 4 weeks of age, which became more pronounced at 12 weeks (Figure 1A, quantification B). Notably, 8-OHdG accumulation was observed not only in TECs lining cysts, but also in non-cystic tubules.

### 2.3. PKD is Associated with Mitochondrial Structural Abnormalities in Tubular and Endothelial Cells

**Figure 2:**
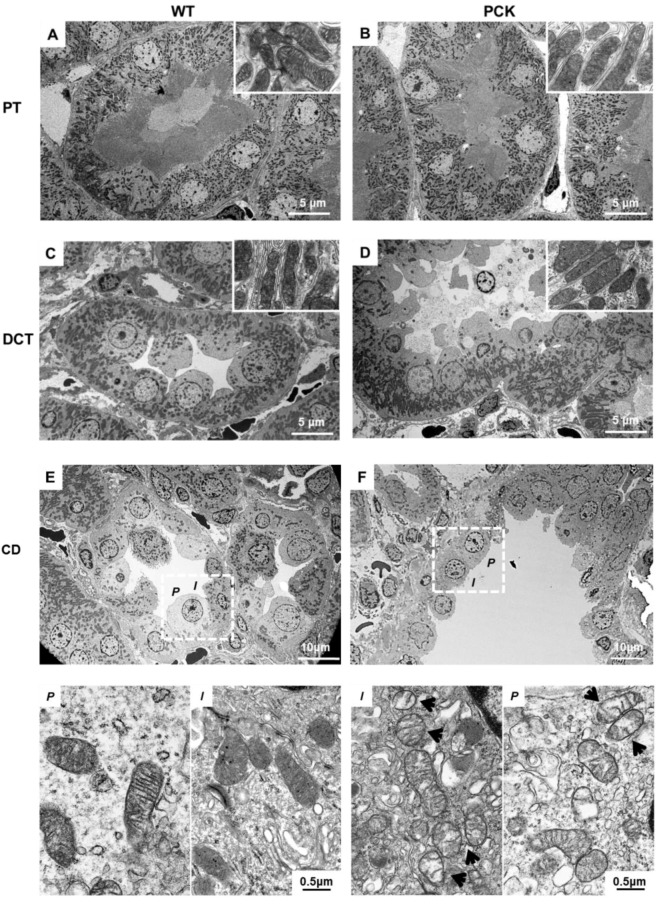
PKD is associated with mitochondrial structural abnormalities in cyst-lining tubular cells. Transmission electron micrograph (TEM) displaying representative tubular structures from WT (left column) and PCK (right column) kidneys at 4 weeks. In PCK kidneys, the cells of proximal tubules (**B**) had normal appearance and were characterized by a tall brush border and extensive invaginations of the basolateral plasma membrane containing abundant elongated mitochondria (×2500 and ×80,000) compared to WT kidneys (**A**). Similarly, the cells from distal tubules in PCK animals (**D**) presented numerous long mitochondria arranged between the foldings of the basal lamina that resembled normal appearing mitochondria as in WT kidneys (**C**). On the other hand, mitochondria from CD principal cells (**P**), characterized by a light appearance, extensive infoldings of the basal plasma membrane, and intercalated cells (**I**), characterized by a denser cytoplasm, numerous apical projections, and more abundant mitochondria, lining micro cysts on PCK animals (**F**), showed cristae remodeling and loss (arrow heads) (×2500 and × 80,000) compared to WT CD (**E**). PT, proximal tubule, DCT, distal convoluted tubule; CD, collecting duct. The panels below E and F are high-magnification images of the *P* and *I* cells in the dotted squares.

We explored tubular mitochondrial structure at different segments of the nephron by transmission electron microscopy (TEM). In PCK animals, mitochondrial morphology was well preserved in all segments of the nephron except for cyst-lining cells, where they showed remodeling and loss of cristae at 4 weeks (Figure 2A–E) and 12 weeks (Supplementary Figure S2). Mitochondrial area and perimeter were preserved at 4 weeks in both principal and intercalated cells of PCK rats and remained unaltered at 12 weeks (Supplementary Figure S3A–D). However, matrix density was lower in principal and intercalated cells of PCK rats at 4 weeks and further decreased at 12 weeks (Supplementary Figure S3E,F). In addition, mitochondria content, assessed by the expression of the mitochondria protein marker translocase of the mitochondrial outer membrane (TOM)-20 and the mitochondrial biogenesis marker peroxisome proliferator activated receptor-γ-coactivator (PGC1)-α, was lower in PCK vs. WT at 4 and 12 weeks (Supplementary Figure S4A–C respectively).

**Figure 3:**
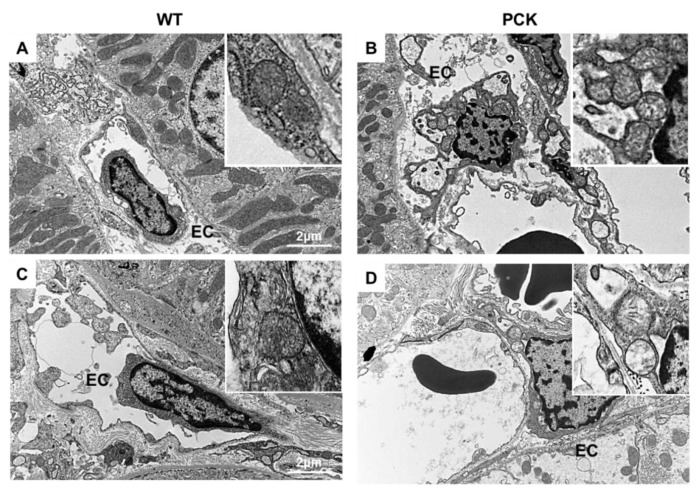
PKD is associated with mitochondrial structural abnormalities in endothelial cells. TEM displaying representative peritubular capillary endothelial cells (EC) from WT (left column) and PCK (right column) kidneys at 4 and 12 weeks ((**A**,**B**) and (**C**,**D**) respectively). In PCK, peritubular capillary EC exhibited morphological abnormalities (cristae remodeling and loss) not only in the capillaries surrounding cystic structures but also in the capillaries surrounding normal appearing tubules (×2500 and ×80,000).

In addition, we examined mitochondrial structure in peritubular EC. Interestingly, EC mitochondrial structural abnormalities (decreased matrix density) not only were present in the pericystic capillaries, but also extended to capillaries in proximity to normal appearing tubules at 4 weeks and 12 weeks (Figure 3A–D).

### 2.4. Renal NOX4 is Upregulated in Tubular Epithelial and Endothelial Cells in PKD 

**Figure 4:**
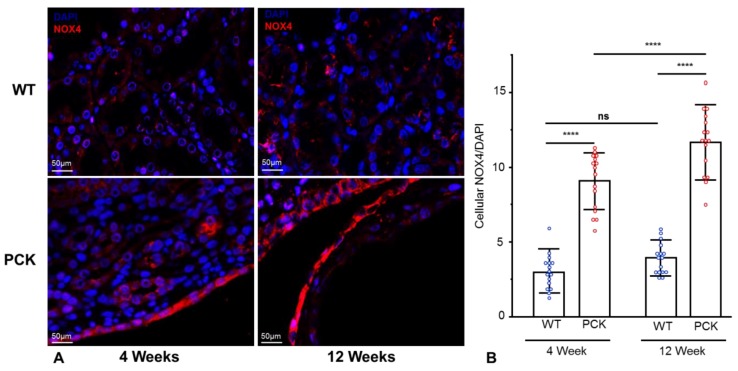
Renal NOX4 is upregulated in tubular epithelial cells in PKD. Representative immunofluorescence staining for NOX4 (red) in renal tissue sections of WT and PCK rats (**A**) and its quantification (**B**), showing increased NOX4 immunoreactivity in PCK vs. WT rats at 4 weeks, which further increased at 12 weeks. **** *p* < 0.0001. NOX4 was quantified as % stained area and adjusted to DAPI-stained % area. (n = 16/group).

To further explore the mechanisms underlying oxidative stress and mitochondrial injury in PKD, we determined NOX4 expression and immunoreactivity at early stages of the disease. To assess NOX4 expression in different sections of the kidney, we performed immunofluorescence staining. At 4 weeks, tubular NOX4 immunoreactivity was increased in PCK compared to WT kidneys and further increased at 12 weeks (Figure 4A and quantification B). Interestingly, NOX4 immunoreactivity increased not only in cyst-lining cells but also in non-cystic tubules (Supplementary Figure S5).

To determine NOX4 expression in different cell compartments, we performed subcellular fractionation and immunoblot analyses. Mitochondria homogenates from PCK kidneys presented a marked increase in NOX4 expression as early as 4 weeks, which became further elevated with disease progression (Supplementary Figure S6A and quantification C). Similarly, nuclear cell lysates from PCK kidneys presented a marked increase in NOX4 expression from early stages, which increased with disease progression (Supplementary Figure S6B and quantification D).

**Figure 5:**
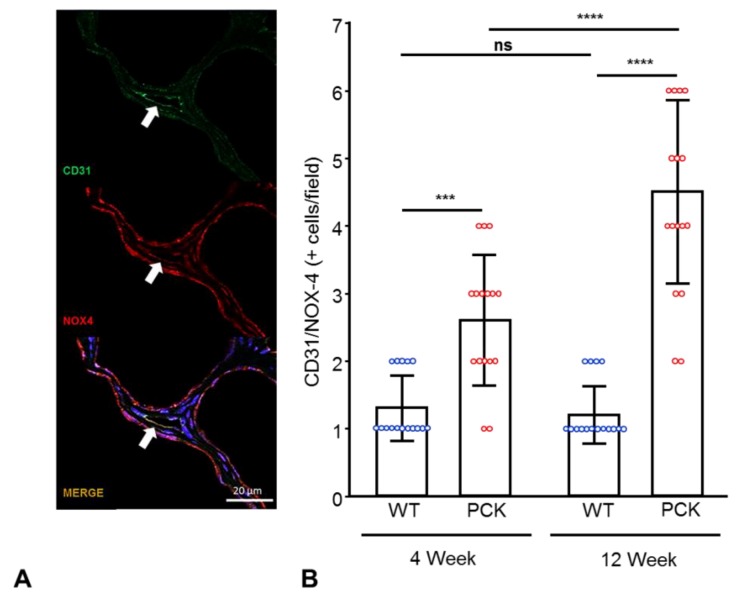
Renal NOX4 is upregulated in peritubular capillary endothelial cells in PKD. Representative IF staining for NOX4 (red) and endothelial cell marker CD31 (green) in renal tissue sections (40×) (**A**) and its quantification (**B**), showing increased number of double-positive cells (NOX4/CD31+) in PCK vs. WT rats at 4 weeks, which further increased at 12 weeks. *** *p* < 0.001; **** *p* < 0.0001. The number of double-positive NOX4/CD31cells was adjusted to the number of positive CD31 cells. (n = 16/group).

We then examined NOX4 in peritubular capillaries by quantifying double-positive (NOX4+/endothelial marker CD31+) cells/field. The number of NOX4+/CD31+ cells was higher in PCK animals at 4 weeks of age and increased further at 12 weeks (Figure 5).

### 2.5. PKD is Associated with Tubular Epithelial Cell Mitochondria Dysfunction

To determine whether NOX4-induced oxidative stress compromises mitochondrial function, we explored mitochondrial respiration in fresh kidney tissue using a polarographic oxygen electrode and found that mitochondrial respiration rate (basal, maximal, and uncoupled respiration) was significantly decreased in PCK rats (Supplementary Figure S7A–D) compared to WT rats at 4 and 12 weeks. However, despite significant reductions in mitochondrial respiratory capacity, PCK rats exhibited higher rates of hydrogen peroxide (H_2_O_2_) emissions from tissue mitochondria, as measured by fluorescence-based monitoring of amplex red oxidation. 

### 2.6. PKD is Associated with Intrarenal Endothelial Dysfunction and Capillary Loss

**Figure 6:**
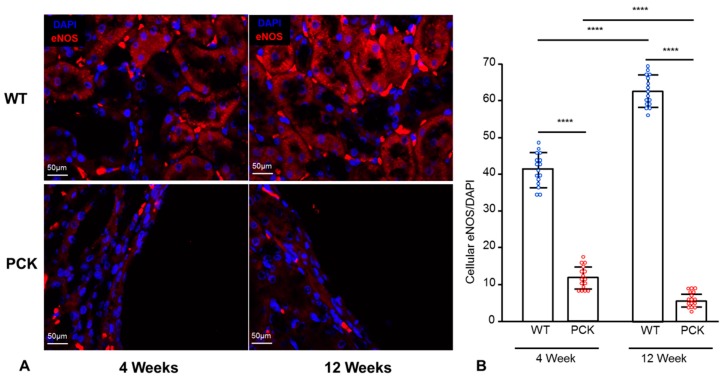
Renal eNOS immunoreactivity is downregulated in PKD. Representative IF staining for eNOS (red) in WT and PCK rats at 4 and 12 weeks (**A**) and its quantification (**B**) showing decreased eNOS immunoreactivity in PCK animals at 4 weeks, which further decreased at 12 weeks. **** *p* < 0.000; eNOS was quantified as % stained area and adjusted to DAPI-stained % area; (n = 16/group).

To assess whether NOX4-induced oxidative stress impairs intrarenal endothelial function, we measured renal immunoreactivity of eNOS, the primary producer of NO in endothelial cells. eNOS immunoreactivity was lower in 4-week-old PCK vs. WT rats and further decreased in 12-week-old PCK rats (Figure 6A and quantification B).

**Figure 7:**
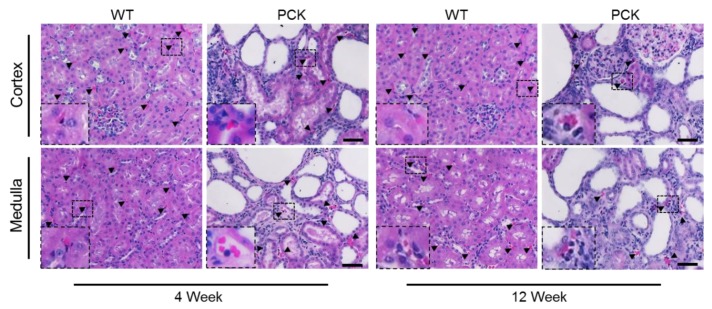
Peritubular capillary loss in PKD. Representative hematoxylin–eosin-stained kidney sections from WT and PCK rats showing capillary loss at 12 weeks. Capillaries were identified by the presence of lumen, red blood cells, and/or an endothelial cell lining (arrowheads), and the ratio of capillary number to non-cystic parenchyma was calculated; (n = 16/group).

Peritubular capillary index, assessed by peritubular capillary count in hematoxylin–eosin (H&E) stained slides and adjusted by cystic area, was not different in the cortex or medulla in PCK vs. WT rats at 4 weeks of age, but it was lower in the cortex and medulla at 12 weeks (Figure 7 and Table 2) and inversely correlated with the cystic index (Supplementary Figure S8). 

## 3. Discussion

In this study, we employed a well-accepted murine model of PKD to explore the mechanisms underlying endothelial dysfunction and renal oxidative stress. We opted for the PCK rat because, despite being orthologous to human autosomal recessive PKD (ARPKD), it has many features that resemble human ADPKD and has been useful for studying its pathogenesis in many preclinical trials for ADPKD [19,20,21,22,23]. Another advantage of rat compared to mouse models is their close physiological similarity to humans, particularly in the cardiovascular and renal systems [24]. 

We found evidence of renal oxidative stress at a very early stage of PKD, reflected by increased renal immunoreactivity of 8-OHdG, that aggravated with disease progression. This is consistent with previous studies in rodent models of PKD that have shown significant increases in 8-OHdG expression in kidney cyst-lining cells [16,25]. Interestingly, we found that this increase in renal 8-OHdG levels was not limited to the cyst-lining TECs and was also present in non-cystic tubules, suggesting that the non-cystic parenchyma is also a contributor to renal oxidative stress. 

The ROS-producing NOX4 is a major source of oxidative stress which is highly expressed in the kidneys, as well as in endothelial and vascular smooth muscle cells [10,11]. In contrast to other NOX isoforms, NOX4 activity is regulated at the expression level, generating predominantly H_2_O_2_ [26]. Elevated NOX4 expression and activity have been reported in a number of renal and cardiovascular diseases, including HTN [13,27,28,29,30,31]. Interestingly, NOX4 downregulation has been shown to restore mitochondrial bioenergetics, reduce mitochondrial ROS production, and attenuate blood pressure response in hypertensive rats [13]. This is consistent with our observations of increased tubular and endothelial NOX4 from early stages of the disease, which exacerbated with disease progression. Importantly, increased NOX4 was not limited to cyst-lining TECs and was present also in non-cystic tubules, suggesting that NOX4 is an important early modulator of cellular ROS. 

The mitochondrial respiratory chain is another major source of ROS, which plays a critical role in damaging cellular components and initiating cell death. Mitochondria are also targets for NOX-derived ROS, and increased expression of mitochondria-localized NOX4 has been shown to increase mitochondrial protein oxidation, leading to decreased electron transport chain (ETC) activity, impaired bioenergetics, and augmented oxidative stress in the vasculature [32]. Moreover, inhibition of NOX4 by genetic or pharmacological approaches has been recently shown to increase ETC activity and result in stimulation of mitochondrial biogenesis [33]. Importantly, ROS generated by mitochondria contribute to mtDNA damage, creating a negative cycle of oxidative stress and mitochondrial dysfunction [34]. Kidneys are rich in mitochondria, which provide the energy necessary to drive tubular function. In addition, mitochondria modulate several cellular functions including proliferation, apoptosis, and intracellular calcium homeostasis. Therefore, it is not surprising that mitochondrial injury and dysfunction have been implicated in the pathogenesis of many kidney diseases including PKD. Indeed, studies in cells and animal models of PKD have reported mitochondrial structural abnormalities and dysfunction, suggesting a key role of this organelle in the disease [16,17,35,36,37,38]. In agreement with this, we found that cyst-lining TECs exhibited significant mitochondrial damage characterized by remodeling and loss of cristae and reflected by decreased matrix density. However, despite substantial evidence of mitochondrial damage and dysfunction, there still remains the question whether these abnormalities are a cause or a consequence of PKD. The current study provides novel evidence of structurally preserved mitochondria in non-cystic TECs both at 4 and 12 weeks. Despite this, we found a decrease in overall renal mitochondria content (TOM-20) and biogenesis (PGC1-α) as early as 4 weeks, in line with previous observations in other murine models of PKD [16]. Furthermore, decreased mitochondrial content was associated with a decrease in global mitochondrial respiration accompanied by increased mitochondrial production of ROS, even when correcting for mitochondrial content. All together, these observations suggest that mitochondrial dysfunction may develop in response to global changes in ROS content and position NOX4 as an important determinant of the cellular redox environment and a contributor to disease progression. 

We also examined the morphology of the mitochondria in renal ECs. Unlike TECs, which possess large numbers of mitochondria, the number of mitochondria in ECs is modest [39]. Nevertheless, EC mitochondria modulate several important pathways that regulate vascular function, including oxidative stress and NO bioavailability [14,15]. We found that as early as 4 weeks, EC mitochondria exhibited important abnormalities (loss of cristae and remodeling). These observations were accompanied by increased NOX4 immunoreactivity, suggesting NOX4-induced EC mitochondrial damage. Importantly, ROS-induced EC mitochondrial abnormalities impaired the endothelial function, as evidenced by decreased eNOS immunoreactivity at 4 weeks and more markedly at 12 weeks. eNOS is the main source of NO in the vascular system and plays an essential role in the regulation of endothelial function. In our study, progressive endothelial dysfunction was associated with capillary loss (decreased capillary index) at 12 weeks and correlated inversely with renal disease progression as assessed by the cystic index. Therefore, NOX4-induced endothelial dysfunction may be an important contributor to vascular pathology and renal disease severity/progression in PKD. 

Our study has several strengths, including its multipronged structural and functional approach that allows studying an early stage of the disease before the development of downstream non-specific pathologic processes, such as inflammation and fibrosis. We acknowledge some limitations of this study, including its descriptive nature and the fact that it considered only two time points. In addition, our functional studies cannot discriminate between individual contributions of ECs and TECs to mitochondria respiration and ROS production, which may warrant further investigation. 

In summary, we found that early PKD is associated with NOX-4-induced oxidative stress and mitochondrial abnormalities predominantly in ECs and TECs lining cysts. Endothelial dysfunction precedes capillary loss, and the latter correlates with worsening of renal disease. Therefore, our observations position NOX4 and EC mitochondria as potential therapeutic targets in PKD. Further mechanistic studies are needed to confirm the role of NOX4 as a main driver of oxidative stress and mitochondria damage in early PKD and whether these results extend to other models of PKD and patients with ADPKD.

## 4. Materials and Methods 

### 4.1. Experimental Design 

All animal procedures were approved by the Institutional Animal Care and Use Committee. All experiments were performed in eight male and eight female PCK and Sprague-Dawley rats (MTPC colony, Mayo Clinic, Rochester, MN), which were studied at 4 and 12 weeks of age. At each time point, blood pressure was determined noninvasively using the tail cuff approach (CODA systems, Kent Scientific), and rats were later placed in metabolic cages to collect 24 h urine samples. Animals were then returned to regular housing and left for 48 h to adjust to the regular environment. All animals were then euthanized by cardiac puncture and exsanguination under anesthesia, and blood and tissues were harvested.

### 4.2. Tissue and Blood Harvest and Analysis

At each time point, the animals were weighed and anesthetized with ketamine (60 mg/kg) and xylazine (10 mg/kg ip). Blood was obtained by cardiac puncture for the determination of serum creatinine and BUN levels. Half of the right kidney was placed into pre-weighed vials containing 10% formaldehyde in phosphate buffer (pH 7.4). These tissues were embedded in paraffin for histological experiments. The other half was preserved in Trump’s fixative for EM analysis. The left kidney was immediately frozen in liquid nitrogen for cell fractionation and protein quantification. 

### 4.3. Histomorphometric Analysis

Longitudinal tissue sections (4 μm) of the kidney were stained with H&E and picrosirius red to determine cystic and fibrotic indexes, respectively. Image analysis procedures were performed with Meta-Morph software (Universal Imaging, West Chester, PA). Digital images were acquired using a light microscope with a high-resolution Nikon Digital camera (Nikon DXM 1200). A colored threshold was applied at a level that separated cysts from non-cystic tissue and picrosirius red-positive material from background to calculate indexes of renal cysts as percentages of total tissue. 

### 4.4. Capillary Index Quantification

Medullary and cortical capillaries were counted at 40× magnification in H&E-stained slides using an ApoTome microscope (Carl ZEISS SMT, Oberkochen, Germany). Capillaries were identified by the presence of lumen, red blood cells, and/or an endothelial cell lining, and the ratio of capillary number to non-cystic parenchyma was calculated.

### 4.5. TEC and EC Mitochondrial Morphology and Function 

Mitochondrial morphology was assessed in TECs and ECs using digital electron microscopy (Phillips CM10 Transmission Electron Microscopy). Renal tissue was preserved in Trump’s fixative solution (4% formaldehyde and 0.1% glutaraldehyde in 0.1 M phosphate buffer) overnight at room temperature, mounted on mesh grids, and stained with aqueous uranyl acetate and lead citrate at the Mayo Clinic’s electron microscopy core facility. For analysis, five representative TECs and ECs were randomly selected, and all their mitochondria examined. Principal and intercalated cells were identified visually by the well-recognized characteristics of cell diameter, shape, and size. Then, mitochondrial area, perimeter, and matrix density were measured in five representative mitochondria in these cells using Image-J (Version 1.5, National Institute of Health [40]), and the results were averaged per mice. Only mitochondria fully contained within the borders of the transmission electron microscopy images were manually traced using the “freehand tool” of ImageJ, which provides mitochondrial area in nanometers squared and mean gray values (brightness). Matrix density was calculated as 1/mean gray values and expressed as arbitrary units.

Mitochondrial oxidative capacity was measured by high-resolution respirometry (Oxygraph 2K, Oroboros) using a stepwise protocol to evaluate various components of the electron transport chain isolated from fresh kidney tissue [41,42]. Oxygen consumption was normalized to tissue wet weight and also to mitochondrial/cell content. Mitochondrial coupling efficiency was measured from the respiratory control ratio (RCR) calculated by the quotient of state-3 and state-4 respiration. Mitochondrial biogenesis was evaluated by renal expression of PGC-1α (Abcam, ab54481, 1/1000) assessed by Western Blot [43]. 

ROS production (H_2_O_2_) of isolated mitochondria was measured with a Fluorolog-3 spectrofluorometer (HORIBA Jobin Yvon) by continuously monitoring oxidation of Amplex Red from fresh tissue. The fluorescent signal was adjusted for background auto-oxidation and calibrated to a standard curve [41,42]. Rates of H_2_O_2_ production were normalized to tissue wet weight and also to mitochondrial/cell content. 

### 4.6. Immunohistology

Renal oxidative stress was determined by quantifying renal immunoreactivity to 8-OHdG (Santa Cruz Biotechnology, Inc., Dallas, TX, USA, sc-130085, 1:200). Results were calculated from the average of 20 images/animals and expressed as stained area % adjusted to DAPI-stained area %. NOX4, eNOS, and TOM-20 immunoreactivity was quantified as above (ab109225, 1:200, BD 610297, 1:100 and sc-11415, 1:200, respectively). To quantify NOX4 in ECs, we performed double staining of CD31 (ab28364, 1:100) and NOX4 (ab109225, 1:200). In each 40× image, the number of double positive NOX4/CD31 cells was counted in 15–20 fields, adjusted by the number of CD31 positive cells, and the results from all fields were averaged. 

### 4.7. Statistical Analysis

Statistical analysis was performed using the JMP Pro 14.0 (SAS) software. Results are expressed as means ± standard deviation. Statistical values were obtained by one-way ANOVA, followed by Tukey post-hoc analysis. Regressions were calculated by the least-squares fit to compare the cortical and medullary peritubular capillary index with the cystic index. Statistical significance for all tests was accepted for *p* < 0.05.

## Figures and Tables

**Table 1:** Animal characteristics and renal function at 4 and 12 weeks in wild-type (WT) and PCK rats.

Animal Characteristic	4 Week		12 Week	
	WT	PCK	WT	PCK
Number of animals	16	16	16	16
Body Weight (g)	152.0 ± 8.4	149.7 ± 7.2	379.9 ± 105.6 ^†^	381.8 ± 91.4 ^†^
KW/BW (%)	1.09 ± 0.08	1.48 ± 0.09 ****	0.79 ± 0.11 ^†^	1.84 ± 0.22 **** ^†^
Kidney Cystic Index (%)	0.05 ± 0.03	15.54 ± 2.92 ****	0.15 ± 0.11	21.4 ± 3.56 **** ^†^
Fibrotic Index (%)	0.40 ± 0.03	0.42 ± 0.04	0.35 ± 0.21	3.51 ± 1.76 **** ^†^
Urine output (24 h, mL)	5.1 ± 0.5	4.8 ± 0.7	12.8 ± 3.3 ^†^	13.2 ± 4.3 ^†^
BUN (mg/dL)	12.3 ± 2.2	12.6 ± 1.9	15.9 ± 1.8 ^‡^	20.1 ± 3.7 **** ^†^
Plasma creatinine (mg/dL)	0.36 ± 0.05	0.35 ± 0.10	0.36 ± 0.10	0.38 ± 0.13
Blood Pressure (mmHg)	109.4 ± 2.3	110.3 ± 2.1	116.2 ± 3.1 ^†^	118.3 ± 2.9 ^†^

Values are means ± SD. KW/BW, kidney weight/body weight, BUN, blood urea nitrogen; **** *p* < 0.0001 significance is against WT; ^‡^ *p* < 0.01, ^†^ *p* < 0.0001, significance is against 4 weeks.

**Table 2:** Capillary index in WT and PCK rats.

Animal Characteristic	4 Week		12 Week	
	WT	PCK	WT	PCK
Number of animals	16	16	16	16
Capillary Index (Cx)	10.9 ± 0.8	10.5 ± 0.6	11.3 ± 0.6	9.3 ± 0.8 **** ^‡^
Capillary Index (Med)	9.4 ± 0.4	9.2 ± 0.5	10.0 ± 0.5 ^+^	8.3 ± 0.5 **** ^†^

Values are means ± SD, Cx, cortex; Med, medulla; **** *p* < 0.0001significance is against WT; ^+^ *p* < 0.01; ^‡^ *p* < 0.001, ^†^ *p* < 0.0001, significance is against 4 weeks.

## Supplementary Materials

The following are available online at https://www.mdpi.com/1422-0067/21/6/1994/s1.

## Abbreviations

ADPKD	Autosomal Dominant Polycystic Kidney Disease
PKD	Polycystic Kidney Disease
ROS	Reactive Oxygen Species
ESRD	End-Stage Kidney Disease
HTN	Hypertension
GFR	Glomerular Filtration Rate
RBF	Renal blood flow
NO	Nitric Oxide
NOX4	NAD(P)H)-oxidase complex-4
WT	Wild-Type (Sprague-Dawley)
