# Supplementary material for: Oxidative Stress and Mitochondrial Abnormalities Contribute to Decreased Endothelial Nitric Oxide Synthase Expression and Renal Disease Progression in Early Experimental Polycystic Kidney Disease
Source: Int J Mol Sci. 2020 Mar 14;21(6):1994. doi: 10.3390/ijms21061994 (PMC7139316; doi:10.3390/ijms21061994)
Supplement: Supplementary file 1 [file ijms-21-01994-s001.pdf]

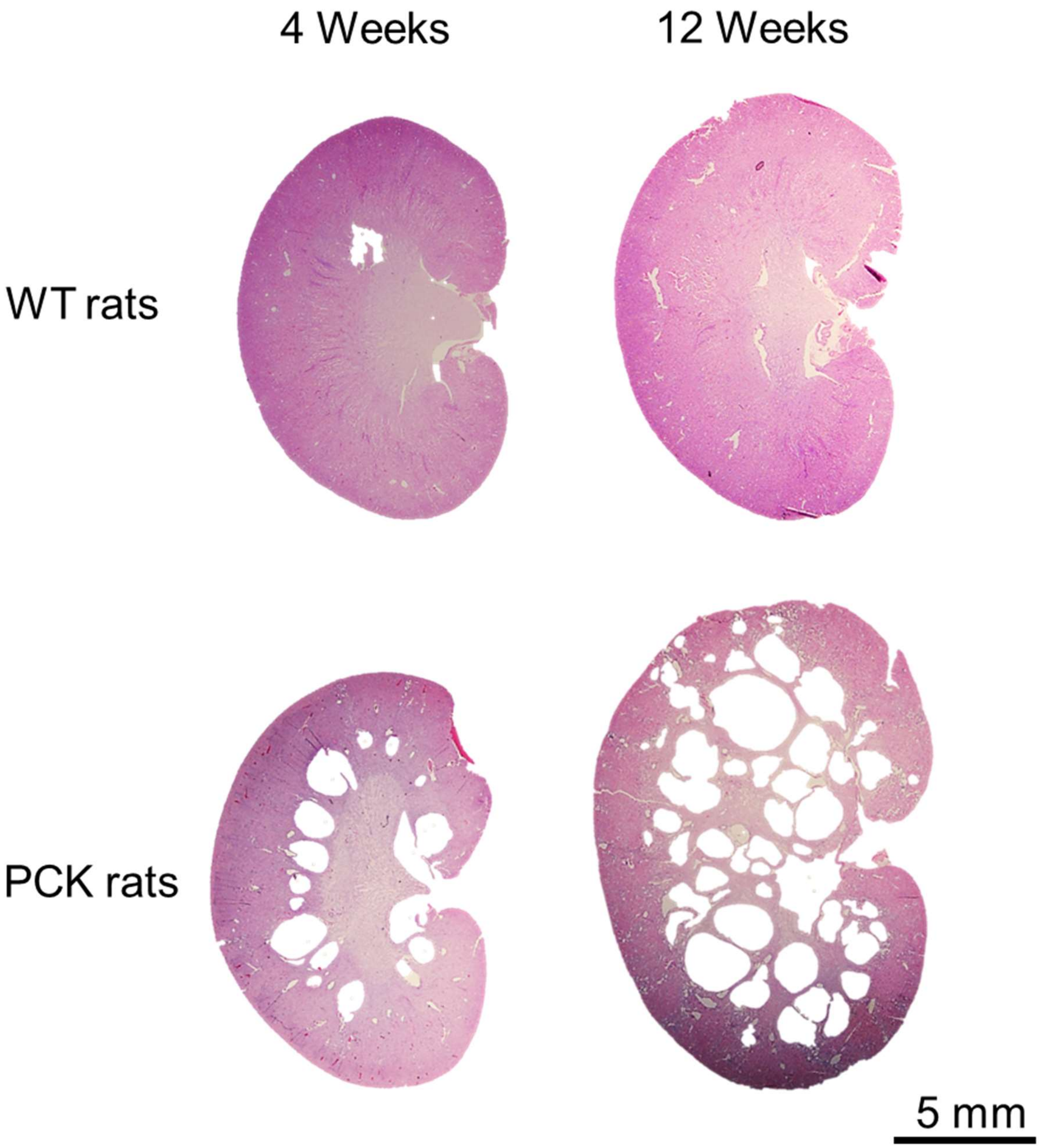

Supplementary Figure 1. Histological images from representative animals from each group. Representative hematoxylin–eosin-stained kidney mid-sections from WT and PCK rats at different ages, showing cyst development predominantly in the medullary region at 4 weeks of age, which expanded to the cortical area by 12 weeks of age in the PCK animals; (n = 16/group).

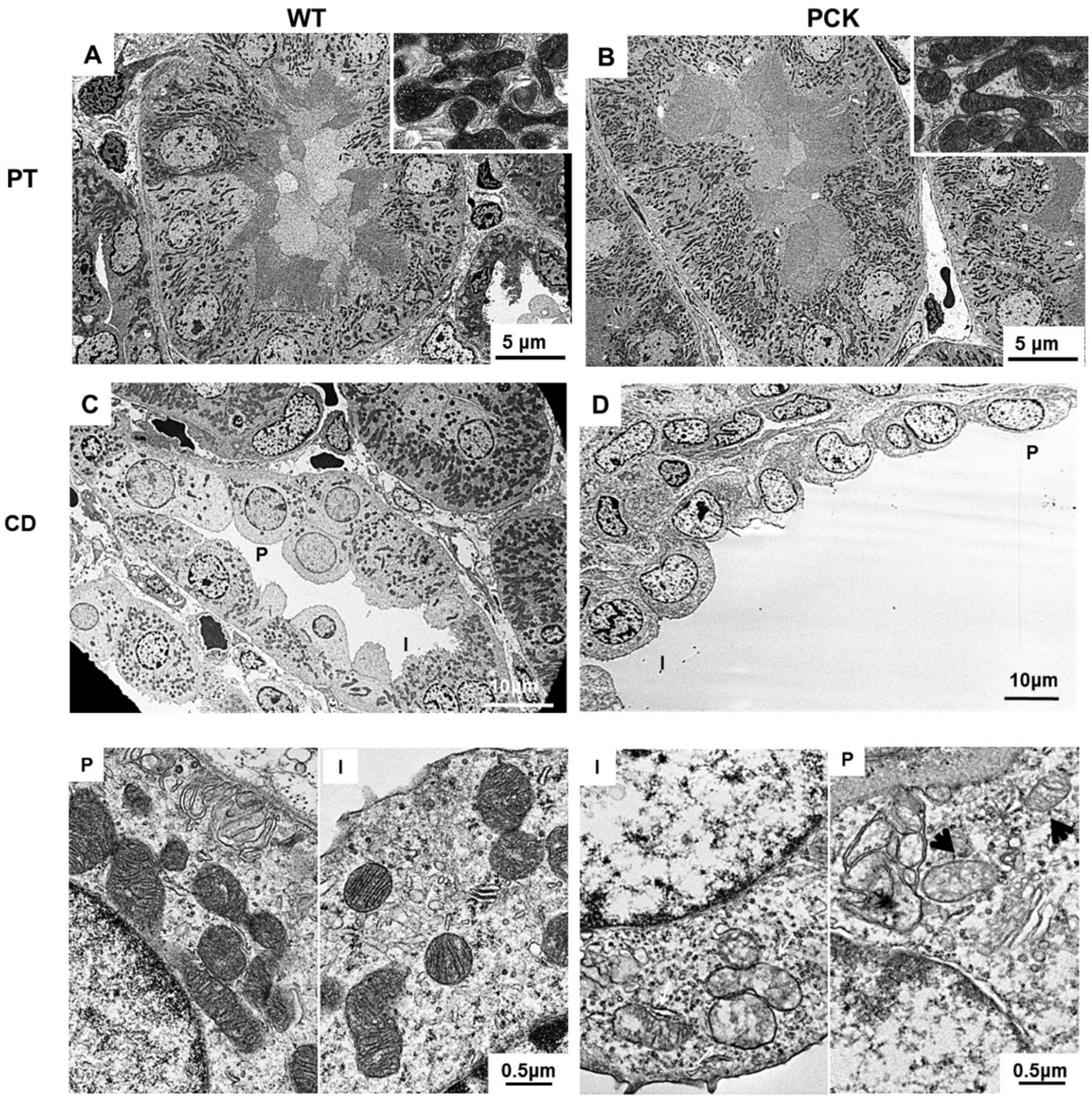

Supplementary Figure 2. PKD is associated with mitochondrial structural abnormalities in cyst-lining tubular cells. Transmission electron micrograph (TEM) displaying representative tubular structures from WT (left column) and PCK (right column) kidneys at 12 weeks. In PCK rats, the cells of the proximal tubules (B) have normal appearance and are characterized by a tall brush border and extensive invaginations of the basolateral plasma membrane and contain abundant elongated mitochondria (×2,500 and x 80,000) compared to WT rats (A). On the other hand, mitochondria from principal (P) and intercalated (I) cells lining the micro cysts in PCK animals (D) show cristae remodeling and loss (arrow heads) (×2,500 and x 80,000). PT, proximal tubule; CD, collecting duct.

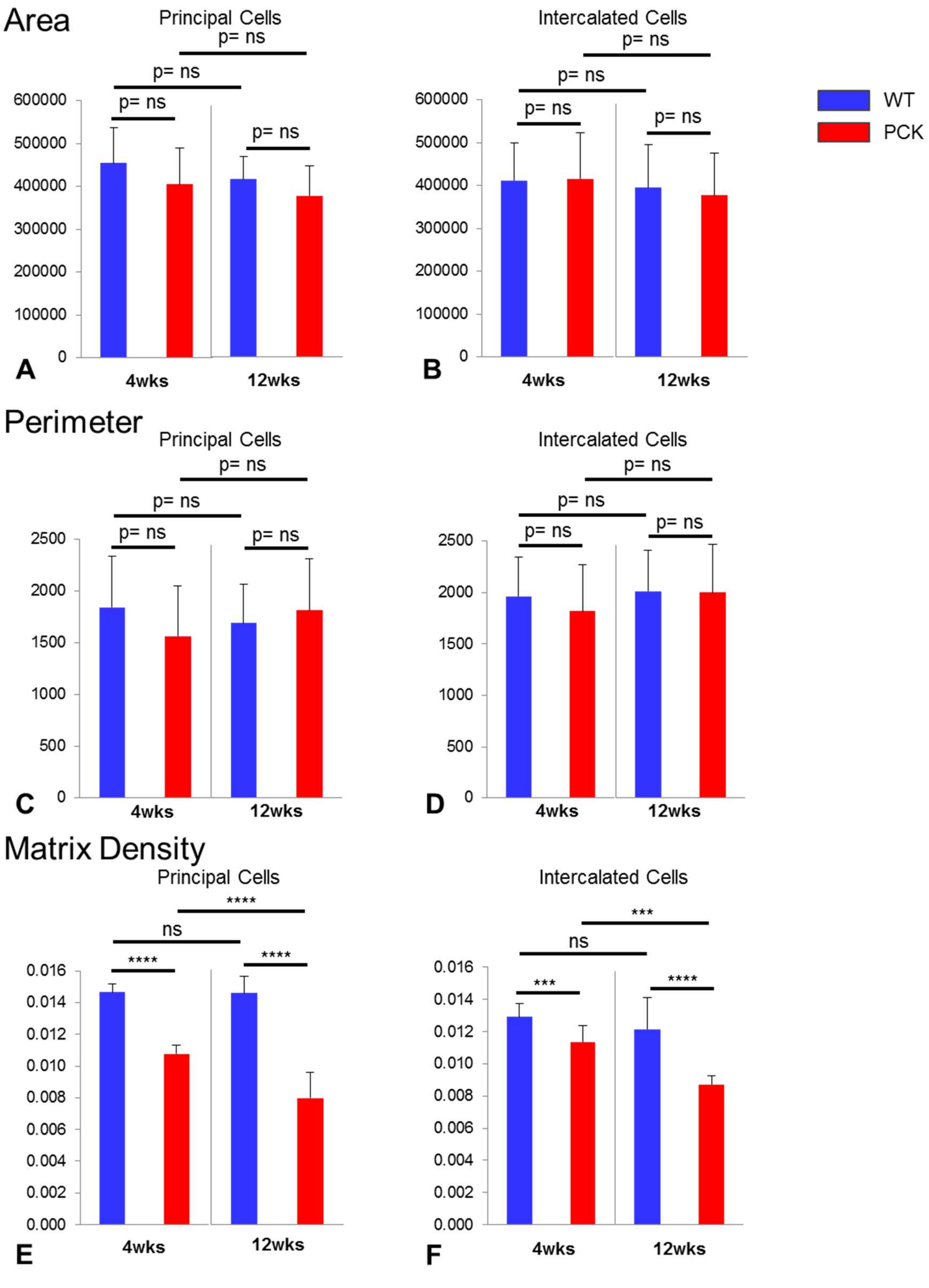

Supplementary Figure 3 PKD is associated with mitochondrial structural abnormalities in cyst-lining tubular sells. Mitochondrial area and perimeter were preserved at 4 weeks of age in both principal and intercalated cells of PCK rats and remained unaltered at 12 weeks (A–D). However, matrix density was lower in principal and intercalated cells of PCK rats at 4 weeks and further decreased at 12 weeks (E–F). *** $p < 0.001$, ****$p < 0.0001$ (n= 16/group).

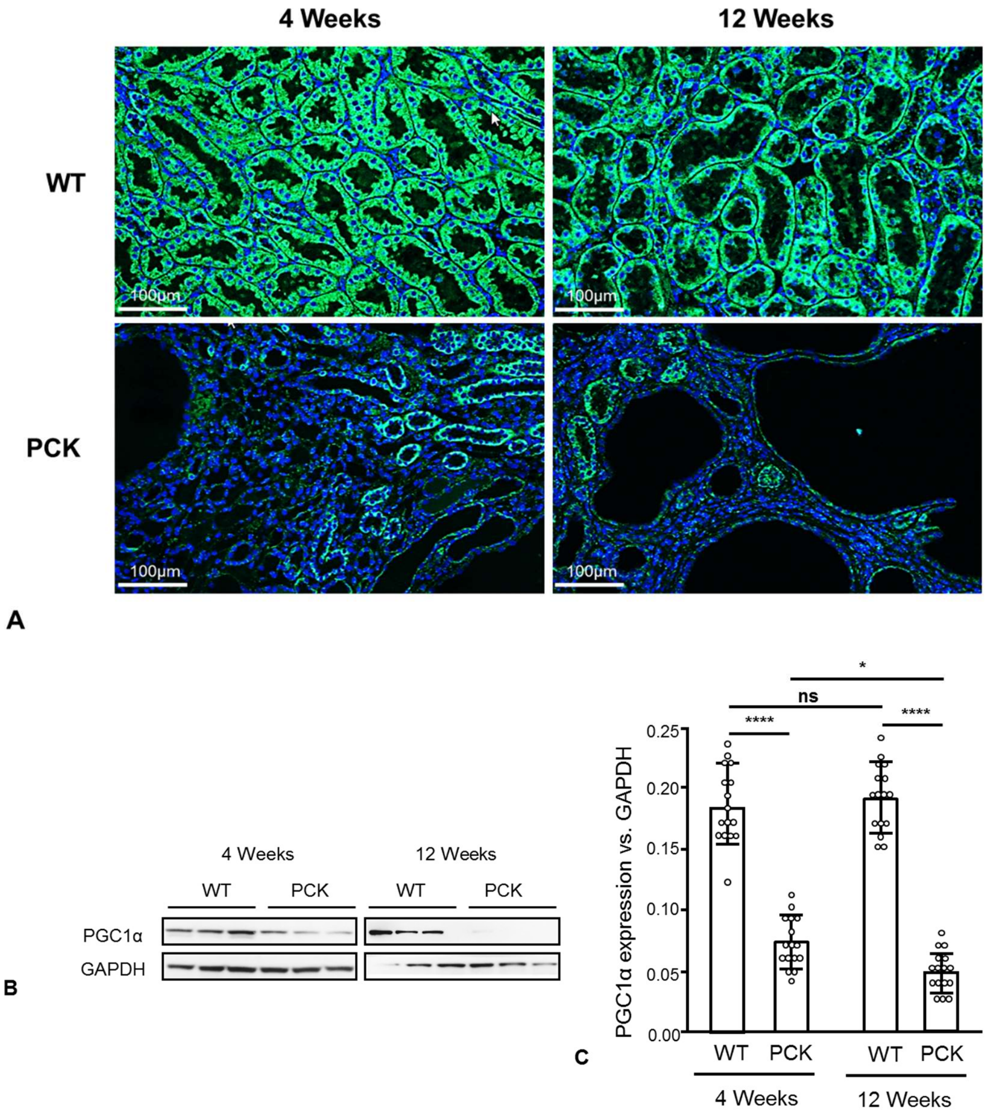

Supplementary Figure 4. Mitochondrial content in WT and PCK rats. Representative IF staining for the mitochondria protein marker translocase of the mitochondrial outer membrane (TOM)-20 at 4 weeks and 12 weeks of age, showing decreased TOM-20 immunoreactivity in PCK vs. WT rats (A). Renal expression of the mitochondrial biogenesis marker peroxisome proliferator activated receptor-γ-coactivator (PGC1)-α was lower in PCK vs. WT rats at 4 and 12 weeks of age (B–C). * $p < 0.05$; **** $p < 0.0001$; (n = 16/group).

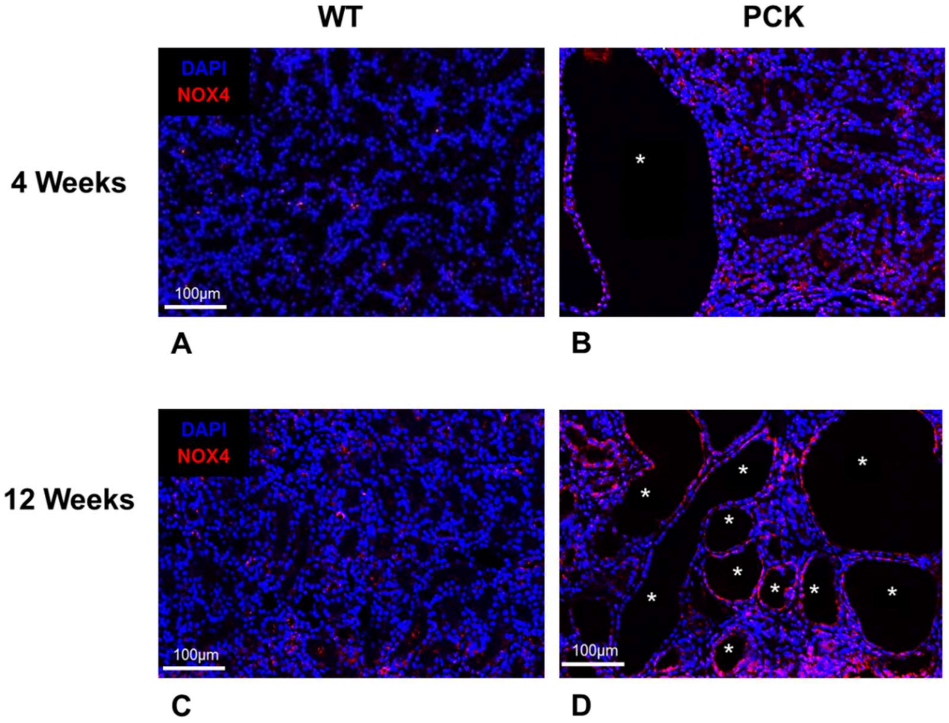

Supplementary Figure 5. Renal tubular immunoreactivity of NOX4 in WT and PCK rats. Representative IF staining for NOX4 showing global increase in NOX4 immunoreactivity in PKD vs. WT rats at 4 weeks of age (A–B). These observations were more pronounced at 12 weeks and were more prominent in the cyst-lining cells (C–D), suggesting that NOX4 is elevated from early stages and increases with disease progression; (n= 16/group).

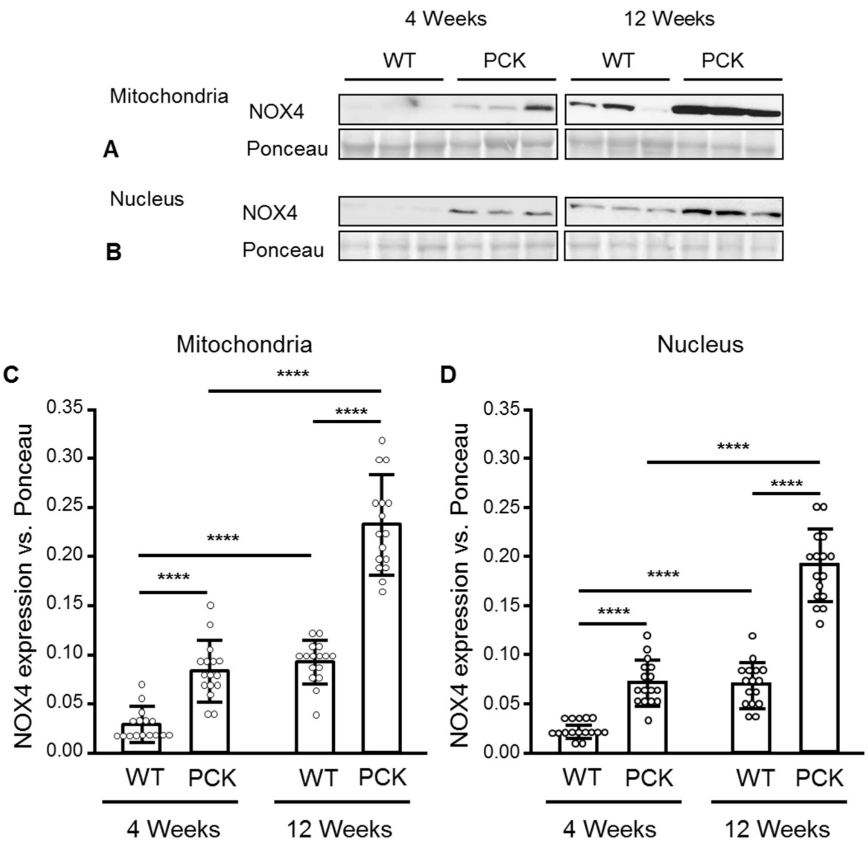

Supplementary Figure 6. Renal NOX4 expression in WT and PCK rats. To determine NOX4 expression in different cell compartments, we performed subcellular fractionation and immunoblot analyses. Mitochondria homogenates (A, C) and nuclear cell lysates (B, D) from PCK kidneys present a marked increase in NOX4 expression as early as 4 weeks, which became further elevated with disease progression. **** $p < 0.0001$; (n= 16/group).

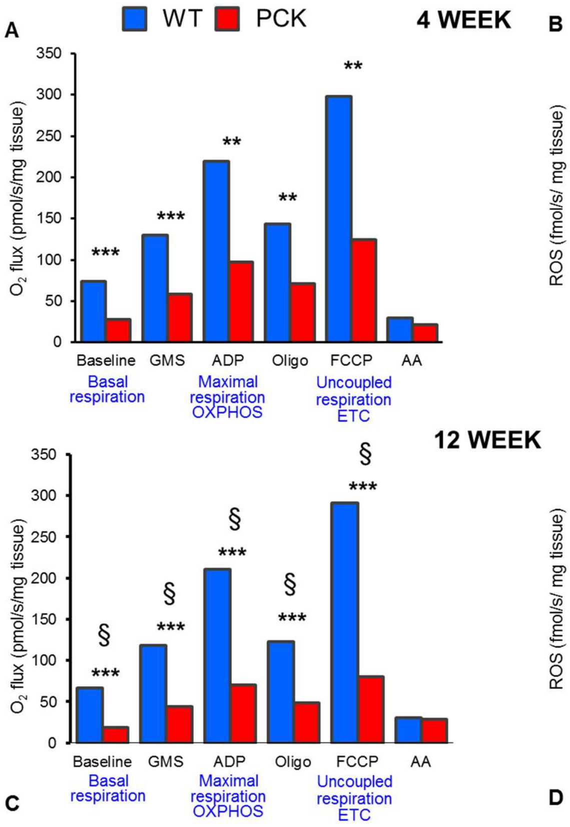

Supplementary Figure 7. Respiration and oxidative stress production in fresh tissue mitochondria in WT and PCK rats at 4 and 12 weeks of age. Mitochondria respiration rate (basal, maximal, and uncoupled respiration) was significantly decreased in PCK compared to WT rats, adjusted by mg of tissue (A) and mitochondrial/cell number (not shown), and decreased further with disease progression ($p < 0.05$) (C). Despite significant reductions in mitochondrial respiratory capacity, PCK rats exhibited higher rates of hydrogen peroxide (H_2O_2) emissions from tissue mitochondria at 4 weeks (B), which increased with disease progression (D). FCCP; carbonyl cyanide-p-trifluoro-methoxy-phenyl-hydrazone, a chemical uncoupler, AA; Antimycin A, complex III inhibitor. GMS, glutamate+malate+succinate, Oligo, oligomycin. * $p < 0.05$, ** $p < 0.01$; *** $p < 0.001$ vs. WT. §$p < 0.05$ vs. 4 weeks.

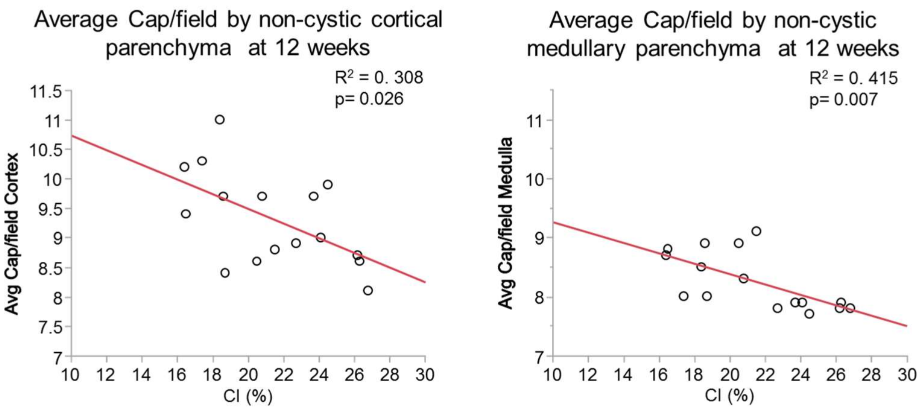

Supplementary Figure 8. Correlation of capillary loss and renal disease progression. Cortical (left) and medullary (right) peritubular capillary index, assessed by peritubular capillary count in H&E-stained slides and adjusted by cystic area, correlated inversely with the cystic index at 12 weeks of age (n= 16).
